# Gene model for the ortholog of *Pten* in *Drosophila miranda*

**DOI:** 10.17912/micropub.biology.000986

**Published:** 2024-09-24

**Authors:** Megan E Lawson, Marjorie Dela Cruz, D'Andrew L. Harrington, Jack A. Vincent, Chelsey McKenna, Anya Goodman, Daron Barnard, Chinmay P. Rele

**Affiliations:** 1 University of Alabama, Tuscaloosa, Alabama, United States; 2 University of Washington Tacoma, Tacoma, United States; 3 College of Southern Nevada, Las Vegas, Nevada, United States; 4 California Polytechnic State University, San Luis Obispo, California, United States; 5 Worcester State University, Worcester, Massachusetts, United States

## Abstract

Gene model for the ortholog of Phosphatase and tensin homolog ( *Pten* ) in the *D. miranda* Apr. 2013 (UC Berkeley DroMir_2.2/DmirGB2) Genome Assembly (GenBank Accession: GCA_000269505.2 ) of *Drosophila miranda* . This ortholog was characterized as part of a developing dataset to study the evolution of the Insulin/insulin-like growth factor signaling pathway (IIS) across the genus *Drosophila* using the Genomics Education Partnership gene annotation protocol for Course-based Undergraduate Research Experiences.

**Figure 1.**
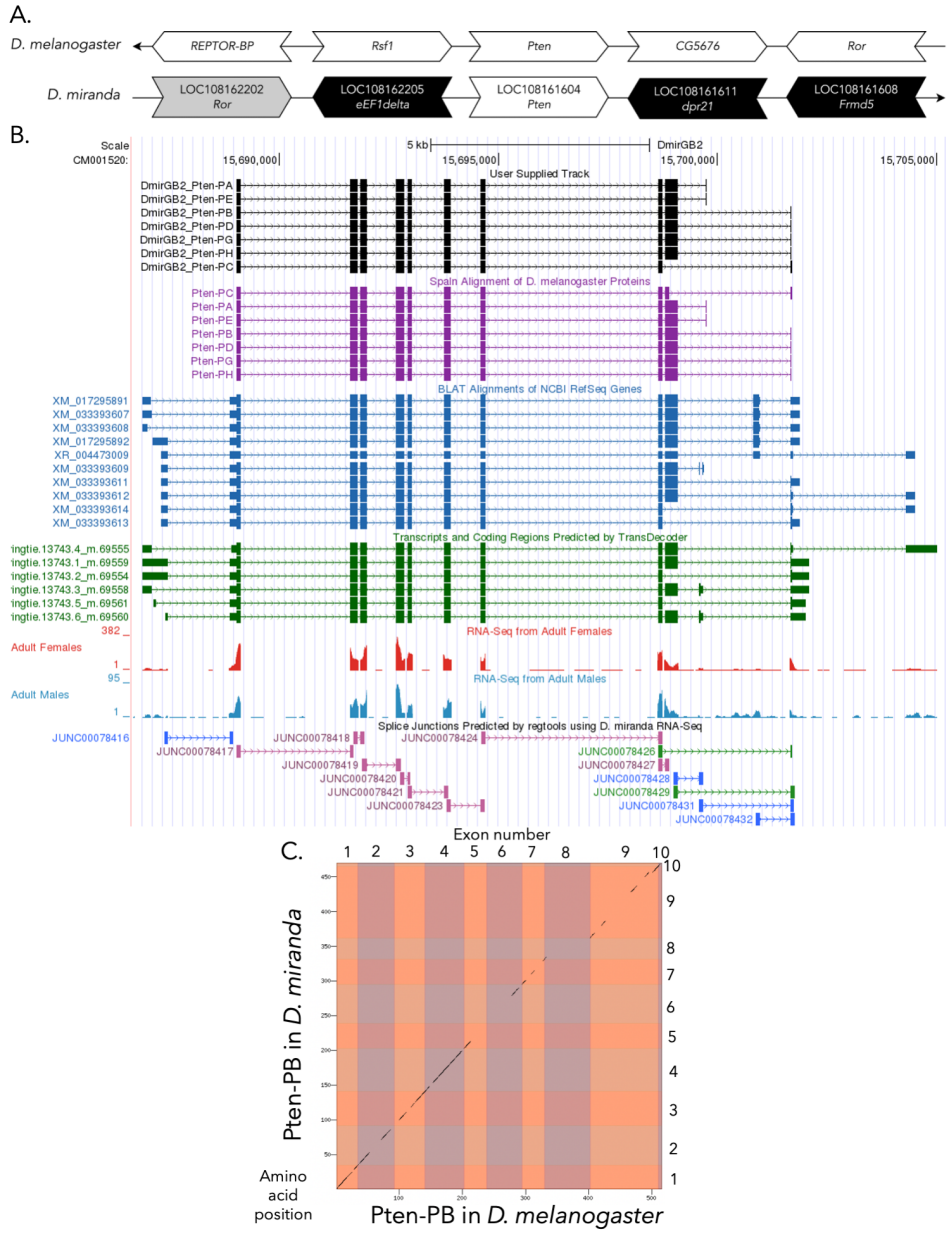
**
(A) Synteny comparison of the genomic neighborhoods for *Pten* in *Drosophila melanogaster* and *D. miranda* .** Thin underlying arrows indicate the DNA strand within which the target gene– *Pten* –is located in *D. melanogaster* (top) and *D. miranda* (bottom). Thin arrow pointing to the right indicates that *Pten* is on the positive (+) strand in *D. miranda* , and thin arrow pointing to the left indicates that *Pten* is on the negative (-) strand in *D. melanogaster* . The wide gene arrows pointing in the same direction as *Pten* are on the same strand relative to the thin underlying arrows, while wide gene arrows pointing in the opposite direction of *Pten* are on the opposite strand relative to the thin underlying arrows. White gene arrows in *D. miranda* indicate orthology to the corresponding gene in *D. melanogaster* , while black gene arrows indicate non-orthology. Gray arrows indicate genes that are present in both genomic neighborhoods, but are not syntenic (in this case, Ror) is upstream of *Pten* in *D. miranda ,* but is downstream of *Pten* in *D. melanogaster .* Gene symbols given in the *D. miranda* gene arrows indicate the orthologous gene in *D. melanogaster* , while the locus identifiers are specific to *D. miranda* . **(B) Gene Model in GEP UCSC Track Data Hub** (Raney et al., 2014). The coding-regions of *Pten* in *D. miranda* are displayed in the User Supplied Track (black); CDSs are depicted by thick rectangles and introns by thin lines with arrows indicating the direction of transcription. Subsequent evidence tracks include Spaln of *D. melanogaster* Proteins (purple, alignment of Ref-Seq proteins from *D. melanogaster* ), BLAT Alignments of NCBI RefSeq Genes (dark blue, alignment of Ref-Seq genes for *D. miranda* ), Transcripts and Coding Regions Predicted by TransDecoder (dark green), RNA-Seq from Adult Females and Adult Males (red and light blue, respectively; alignment of Illumina RNA-Seq reads from *D. miranda* ), and Splice Junctions Predicted by regtools using *D. miranda* RNA-Seq ( SRP009365 ). Splice junctions shown have a minimum read-depth of 10 with 10-49, 50-99, and 100-499 supporting reads in blue, green, and pink, respectively. **(C) Dot Plot of Pten-PB in *D. melanogaster* ( *x* -axis) vs. the orthologous peptide in *D. miranda* ( *y* -axis).** Amino acid number is indicated along the left and bottom; CDS number is indicated along the top and right, and CDSs are also highlighted with alternating colors. The gaps in the dot plot indicate regions with low sequence similarity.

## Description

**Table:** 


* This article reports a predicted gene model generated by undergraduate work using a structured gene model annotation protocol defined by the Genomics Education Partnership (GEP; thegep.org ) for Course-based Undergraduate Research Experience (CURE). The following information in this box may be repeated in other articles submitted by participants using the same GEP CURE protocol for annotating Drosophila species orthologs of Drosophila melanogaster genes in the insulin signaling pathway. * "In this GEP CURE protocol students use web-based tools to manually annotate genes in non-model *Drosophila* species based on orthology to genes in the well-annotated model organism fruitfly *Drosophila melanogaster* . The GEP uses web-based tools to allow undergraduates to participate in course-based research by generating manual annotations of genes in non-model species (Rele et al., 2023) . Computational-based gene predictions in any organism are often improved by careful manual annotation and curation, allowing for more accurate analyses of gene and genome evolution (Mudge and Harrow 2016; Tello-Ruiz et al., 2019) . These models of orthologous genes across species, such as the one presented here, then provide a reliable basis for further evolutionary genomic analyses when made available to the scientific community.” (Myers et al., 2024) . “The particular gene ortholog described here was characterized as part of a developing dataset to study the evolution of the Insulin/insulin-like growth factor signaling pathway (IIS) across the genus *Drosophila* . The Insulin/insulin-like growth factor signaling pathway (IIS) is a highly conserved signaling pathway in animals and is central to mediating organismal responses to nutrients (Hietakangas and Cohen 2009; Grewal 2009) .” (Myers et al., 2024) .

We propose a gene model for the *D. miranda* ortholog of the *D. melanogaster* *Pten* gene. The genomic region of the ortholog corresponds to the uncharacterized protein LOC108161604 (RefSeq accession XP_033249503.1 ) in the Apr. 2013 (UC Berkeley DroMir_2.2/DmirGB2) Genome Assembly of *D. miranda* ( GCA_000269505.2 ). This model is based on RNA-Seq data from *D. miranda* ( SRP009365 ) and *Pten* in *D. melanogaster* using FlyBase release FB2023_02 ( GCA_000001215.4 ; Larkin et al., 2021; Gramates et al., 2022).

The *Drosophila Phosphatase and tensin homolog* ( *Pten* also known as *dPTEN* ; FBgn0026379), identified due to is conservation to the human tumor suppressor gene acts as a protein and lipid phosphatase in the insulin signaling pathway (Goberdhan et al., 1999; Huang et al., 1999) . Pten is known to affect cell number and size through the inhibition of the phosphoinositide 3-kinase (PI3K) and AKT kinase pathways (Goberdhan et al., 1999; Gao et al., 2000) . Pten is involved in stabilizing cell junctions (Bardet et al., 2013) and regulates the cytoskeleton, controlling the localization and organization of actin (Goberdhan et al., 1999; von Stein et al., 2005) .

*D. miranda* (NCBI:txid 7229) is part of the *pseudoobscura* species subgroup within the *obscura* species group in the subgenus *Sophophora* of the genus *Drosophila* (Sturtevant 1942; Buzzati-Traverso and Scossiroli 1955) . It was first described by Dobzhansky in 1935. Like other members of the species subgroup, it is endemic to the New World distributed through Canada, the USA, and Mexico, sympatric with its sibling species *D. pseudoobscura* (Markow and O'Grady 2006), living in temperate forest environments.

***Synteny***

The reference gene, *Pten ,* occurs on chromosome chr2L in *D. melanogaster* and is flanked upstream by *Repressor splicing factor 1* ( *Rsf1* ) and *REPTOR-binding partner* ( *REPTOR-BP* ) and downstream by *CG5676* and *Ror* ( *Ror* ). The *tblastn* search of *D. melanogaster* Pten-PB against the *D. miranda* ( GCA_000269505.2 ) Genome Assembly (database) placed the putative ortholog of *Pten* within scaffold CM001520 (CM001520.2) at locus LOC108161604 ( XP_033249503.1 )— with an E-value of 1e-36 and a percent identity of 86.15%. Furthermore, the putative ortholog is flanked upstream by LOC108162205 ( XP_017152305.1 ) and LOC108162202 ( XP_017152302.2 ), which correspond to *eukaryotic translation elongation factor 1 delta* ( *eEF1delta* ) and *Ror* in *D. melanogaster* (E-value: 2e-103 and 0.0; identity: 65.25% and 84.53%, respectively, as determined by *blastp* ; Figure 1A, A ltschul et al., 1990). The putative ortholog of *Pten* is flanked downstream by LOC108161611 ( XP_017151399.1 ) and LOC108161608 ( XP_017151393.1 ), which correspond to *defective proboscis extension response 21* ( *dpr21* ) and *FERM domain containing* ( *Frmd5* ) in *D. melanogaster* (E-value: 4e-156 and 0.0; identity: 76.51% and 75.64%, respectively, as determined by *blastp* ). The putative ortholog assignment for *Pten* in *D. miranda* is supported by the following evidence: The *tblastn* results are of very good quality, and all isoforms and CDSs present in *D. melanogaster* appear to be present in *D. miranda* as well. While local synteny is not well-conserved in this neighborhood, this is likely still the correct ortholog to *Pten* in *D. miranda .* Additionally, while the nearby *Ror* gene is not syntenic across the two neighborhoods, its proximity to *Pten* in both species is further evidence that this is the correct ortholog assignment for *Pten* in *D. miranda .*

***Protein Model***

*Pten* in *D. miranda* has seven protein coding isoforms, Pten-PA, Pten-PB, Pten-PC, Pten-PD, Pten-PE, Pten-PG, Pten-PH ( Figure 1B ). Protein isoforms Pten-PA and Pten-PE are identical, and are encoded by ten CDSs in the genome and translated from mRNAs Pten-RA and Pten-RE (which differ in their UTRs). Protein isoforms Pten-PB, Pten-PD, Pten-PG, and Pten-PH are identical, and are encoded by ten CDSs in the genome and translated from mRNAs Pten-RB, Pten-RD, Pten-RG, and Pten-RH (which differ in their UTRs). Protein isoform Pten-PC is encoded by nine CDSs in the genome, and translated from mRNA Pten-RC. Relative to the ortholog in *D. melanogaster* , the CDS number is conserved, as *D. melanogaster* also has seven mRNA isoforms, which translate into three unique protein sequences, and two encoded by ten CDSs and one encoded by nine CDSs. The sequence of Pten-PB in *D. miranda* has 64.99% identity (E-value: 0.0) with the protein-coding isoform Pten-PB in *D. melanogaster* , as determined by *blastp* ( Figure 1C ). Some regions of low sequence similarity exist particularly in CDSs five through ten, with CDS eight being substantially shorter in the target gene relative to the reference gene. Coordinates of this curated gene model are stored by NCBI at GenBank/BankIt (accession BK064496 , BK064497 , BK064498 , BK064499 , BK064500 , BK064501 , BK064502 **)** . These data are also archived in the CaltechDATA repository (see “Extended Data” section below).

***Special characteristics of the protein model***

**Lack of Synteny** : The genomic neighborhood of *Pten* in *D. melanogaster* is not syntenic to the neighborhood of the *Pten* ortholog in *D. miranda .* However, due to the high-quality matches from the blast searches of the target gene itself, as well as the high conservation of all isoforms and CDSs, this is likely still the correct ortholog of *Pten* in *D. miranda ,* and the lack of synteny is likely due to expected divergence between the two species.

## Methods

Detailed methods including algorithms, database versions, and citations for the complete annotation process can be found in Rele et al. (2023). Briefly, students use the GEP instance of the UCSC Genome Browser v.435 ( https://gander.wustl.edu ; Kent WJ et al., 2002; Navarro Gonzalez et al., 2021) to examine the genomic neighborhood of their reference IIS gene in the *D. melanogaster* genome assembly (Aug. 2014; BDGP Release 6 + ISO1 MT/dm6). Students then retrieve the protein sequence for the *D. melanogaster* target gene for a given isoform and run it using *tblastn* against their target *Drosophila* species genome assembly [ *D. miranda* ( GCA_000269505.2 )] on the NCBI BLAST server ( https://blast.ncbi.nlm.nih.gov/Blast.cgi , Altschul et al., 1990) to identify potential orthologs. To validate the potential ortholog, students compare the local genomic neighborhood of their potential ortholog with the genomic neighborhood of their reference gene in *D. melanogaster* . This local synteny analysis includes at minimum the two upstream and downstream genes relative to their putative ortholog. They also explore other sets of genomic evidence using multiple alignment tracks in the Genome Browser, including BLAT alignments of RefSeq Genes, Spaln alignment of *D. melanogaster* proteins, multiple gene prediction tracks (e.g., GeMoMa, Geneid, Augustus), and modENCODE RNA-Seq from the target species. Genomic structure information (e.g., CDSs, CDS number and boundaries, number of isoforms) for the *D. melanogaster* reference gene is retrieved through the Gene Record Finder ( https://gander.wustl.edu/~wilson/dmelgenerecord/index.html ; Rele et al *.,* 2023). Approximate splice sites within the target gene are determined using *tblastn* using the CDSs from the *D. melanogaster* reference gene. Coordinates of CDSs are then refined by examining aligned modENCODE RNA-Seq data, and by applying paradigms of molecular biology such as identifying canonical splice site sequences and ensuring the maintenance of an open reading frame across hypothesized splice sites. Students then confirm the biological validity of their target gene model using the Gene Model Checker ( https://gander.wustl.edu/~wilson/dmelgenerecord/index.html ; Rele et al., 2023), which compares the structure and translated sequence from their hypothesized target gene model against the *D. melanogaster* reference gene model. At least two independent models for each gene are generated by students under mentorship of their faculty course instructors. These models are then reconciled by a third independent researcher mentored by the project leaders to produce a final model like the one presented here. Note: comparison of 5' and 3' UTR sequence information is not included in this GEP CURE protocol.

## Data Availability

Description: A GFF, FASTA, and PEP of the model. Resource Type: Model. DOI: https://doi.org/10.22002/fayvg-4kv33
